# Increased self-regulation of eating behavior is associated with reduced generalized anxiety disorder in Saudi Arabia

**DOI:** 10.3389/fpsyg.2024.1480812

**Published:** 2024-10-10

**Authors:** Mai Adil Ghabashi

**Affiliations:** Department of Clinical Nutrition, Faculty of Applied Medical Sciences, Umm Al-Qura University, Makkah, Saudi Arabia

**Keywords:** self-regulation, eating behavior, generalized anxiety disorder, mental health, dietary habits, Kingdom of Saudi Arabia

## Abstract

**Introduction:**

There is a growing body of evidence suggesting that self-regulation of eating behavior (SREB) is closely linked to dietary habits and mental health. However, little is known about these relationships within the Saudi Arabian population. This study aimed to address this gap.

**Methods:**

A digital cross-sectional study was conducted utilizing the SREB and generalized anxiety disorder (GAD-7) questionnaires, along with a brief survey about dietary habits. Linear and logistic regression analyses were employed to examine these associations, with a total of 651 Saudi participants enrolled in the study.

**Results:**

The results showed that only 15.5% of participants demonstrated a high capacity for SREB, while 65% experienced moderate or severe symptoms of GAD. Greater SREB was significantly associated with reduced GAD scores (β = −0.13, 95% CI; −0.12 to −0.03; *p* < 0.001) and lower BMI (β = −0.10, 95% CI; 0.08 to −0.00; *p* = 0.01). Additionally, predictive factors for high capacity for SREB included higher daily intake of fruits (OR = 2.90, 95% CI; 1.44 to 5.84; *p* = 0.003) and regular breakfast consumption (OR = 1.64, 95% CI; 1.01 to 2.63; *p* = 0.04).

**Discussion:**

These findings suggest that enhancing SREB may be a promising strategy for obesity prevention and improving mental health outcomes among the Saudi population. Implementing interventions aimed at promoting healthier dietary habits could support the physical and mental well-being of this population.

## 1 Introduction

Self-regulation of eating behavior (SREB) refers to an individual's ability to control and manage their food intake and eating patterns (Johnson et al., 2012; Kliemann et al., 2016). SREB involves cognitive, emotional, and behavioral processes that allow people to initiate, maintain, and modify their eating behaviors according to their personal goals and values (Godet et al., 2022). SREB is particularly important in the Kingdom of Saudi Arabia (KSA), where obesity has reached epidemic levels. According to the latest national report published in 2023, approximately 40% of Saudi adults were with overweight, and an additional 24% were with obesity (General Authority for Statistics, 2024). This places considerable demands on the economic system, with the cost of medical care reaching $19 billion per year (Okunogbe et al., 2021). The increasing rates of overweight and obesity in the KSA is likely attributed to the concurrent occurrence of the nutrition transition, coinciding with the advent of oil discovery since the 1960s (Popkin, 2002). This transition involves the processes of urbanization, the adoption of sedentary lifestyles, and alterations in dietary habits (Musaiger et al., 2012). It is suggested that improved SREB may help in addressing the obesity epidemic (Chew et al., 2022). This is likely attributed to the fact that the high capacity for SREB is linked to appetite control. Enhancing SREB can help individuals better recognize and respond to internal hunger and satiety cues, leading to the prevention of calorie overconsumption.

In addition, it is well documented in the literature that less healthy dietary habits are associated with several chronic diseases such as diabetes and hypertension (Tejani et al., 2023; Shimbo, 2016). Unfortunately, type 2 diabetes is prevalent among 17.7% of the Saudi population (International Diabetes Federation, 2024) while 9% are affected by hypertension (Lee Batisky et al., 2023). Conversely, healthy dietary habits could help in prevention and management of chronic disease (Jayedi et al., 2020). For instance, a high consumption of fruits and vegetables has been shown to have a beneficial impact on glycemic control and blood pressure regulation in patients with diabetes and hypertension (Chandalia et al., 2000; Tejani et al., 2023). This is likely attributable to the fiber, micronutrient, and antioxidant content of these plant-based foods. Unfortunately, fruits and vegetables consumption found to be insufficient among 94% of the Saudi population (Ministry of Health, 2019). Within this context, improved SREB may play a role in improving dietary habits and thereby reducing the burden of chronic disease in the KSA. That is, previous research found that high SREB was associated with better dietary habits including the higher consumption of fruits and vegetables (Stadler et al., 2010).

Moreover, the Saudi National Mental Health Survey indicated that mental disorders are prevalent in Saudi Arabia, with an overall prevalence rate of 20.2%. Of these mental health conditions, anxiety was found to be the most common, affecting 12.3% of the population (Altwaijri et al., 2020). Interestingly, there is emerging evidence that healthy dietary habits could be associated with better mental health. For instance, a systematic review that included 61 studies reported a favorable correlation between the consumption of vegetables and fruits with psychological wellbeing. That is, the higher consumption of vegetables and fruits, especially, was linked with a heightened levels of optimism as well as a reduction in psychological distress (Głabska et al., 2020). Conversely, less healthy dietary habits could be associated with adverse impacts on psychological wellbeing. For example, a systematic review and meta-analysis that involved 14 studies found that skipping breakfast was associated with psychological distress and depression (Zahedi et al., 2022).

While some relationships have begun to emerge, as previously indicated, numerous aspects of this field remain under-researched due to limited research. Therefore, there is a need to gain a deeper understanding of how self-regulation in eating habits, dietary choices, and mental health are interconnected. This area presents a promising domain for empirical investigations that could yield valuable insights into the complex relationships among these factors. The findings of such investigations could then inform the development of more comprehensive approaches to promoting both Psychological and physiological wellbeing wellness.

Unfortunately, there is a scarcity of studies delving into the self-regulation of eating behavior in the KSA. Merely one study has been undertaken in this field, indicating that only 17.7% of the Saudi population exhibits a high degree of self-regulation in their eating behaviors. Additionally, it was reported that SREB was correlated with chronotype. Specifically, it was found that individuals who are morning-oriented tend to exhibit a higher level of SREB compared to their evening-oriented counterparts (Al-Hazmi and Noorwali, 2023). Nevertheless, the associations between SREB and other variables including mental health aspects and dietary habits have not yet been investigated among the Saudi Arabian community. Therefore, this study aims to address this gap in the literature by exploring such associations. In particular, the primary aim of the present study is to examine the factors associated with a high capacity for self-regulation of eating behavior. This is crucial for understanding the factors associated with self-regulation of eating behavior among the Saudi population which can provide valuable insights into the development of targeted strategies. This would help informing interventions that aim to promote healthy eating practices and overall wellbeing in the KSA.

## 2 Materials and methods

### 2.1 Study design

A cross-sectional investigation was carried out utilizing an anonymous online questionnaire administered through Google Forms. The questionnaire was disseminated employing the snowball sampling technique, leveraging social media platforms for distribution purposes. Before engaging in the survey, participants were explicitly informed, via the digital interface, of the voluntary nature of their participation and the assurance of anonymity, with no collection of personally identifiable information. Participants were guaranteed that all data handling and analysis would be treated with strict confidentiality. Only those individuals who provided informed consent proceeded to the main sections of the questionnaire. This approach allowed for the recruitment of a diverse sample while protecting the privacy and anonymity of all participants throughout the data collection process.

### 2.2 Inclusion and exclusion criteria

#### 2.2.1 Inclusion criteria

Adults aged 18 years and above.

Saudi Arabian nationality.

Healthy individuals (not experiencing any physical or mental health issues).

Not pregnant.

Demonstrated desire to maintain healthy dietary habits.

#### 2.2.2 Exclusion criteria

Individuals younger than the target age range.

Non-Saudi participants.

Participants with pre-existing mental or medical conditions.

Pregnant women.

Absence of the desire to adhere to a healthy diet.

### 2.3 Sample size

The required sample size was calculated using the RaoSoft digital sample size calculator. This determination indicated that a minimum of 377 participants were needed to conduct the study with a 95% confidence interval and 5% margin of error. However, the final sample size exceeded this target, with 1,069 individuals completing the questionnaire. Of these respondents, 418 were excluded as they did not meet the established inclusion criteria and were excluded in the data analysis.

### 2.4 Data collection

The data collection process involved gathering information on basic sociodemographic factors such as sex, age and education. Additionally, the study participants self-reported their anthropometric measurements, including weight and height. Body mass index (BMI) was subsequently calculated as weight in kilograms divided by height in meters squared. participant weight status was then categorized according to standard BMI thresholds, with underweight defined as BMI < 18.5 kg/m^2^, normal weight as 18.5 to 24.9 kg/m^2^, overweight as 25.0 to 29.9 kg/m^2^, and obesity as 30.0 kg/m^2^ or greater. Moreover, data collection included assessing participants' self-regulation of eating behavior as well as various variables associated with psychological wellbeing. Further details regarding this process are provided below.

### 2.5 Self-regulation of eating behavior

The self-regulation of eating behavior was assessed using the 5-item short form of the Self-Regulation of Eating Behavior Questionnaire (SREBQ-5) (Kliemann et al., 2016). The SREBQ-5 was translated into Arabic and validated as part of a recent study published in 2023 which was conducted in the KSA (Al-Hazmi and Noorwali, 2023). The SREBQ evaluates the extent to which individuals can regulate and control their eating behaviors in order to achieve and/or maintain their dietary goals. The SREBQ-5 began with a list of 13 commonly-craved foods, and participants were asked if they intend to control their consumption of these tempting foods and if they have the intention to follow a healthy diet. These questions are designated to exclude participants who do not have healthy dietary intentions by any chance from the survey. Hence, their responses will be excluded during the data analysis process. The SREBQ-5 items were scored on a 5-point Likert scale from 1 (strongly disagree) to 5 (strongly agree), with higher total scores reflecting greater self-regulation of eating behavior. Total mean SREBQ-5 scores were categorized as low (<2.8), moderate (2.8–3.6), or high (>3.6) self-regulation, based on the established cut-offs (Kliemann et al., 2016).

### 2.6 General anxiety disorder

Assessing GAD was performed using a validated questionnaire consisting of seven items (Spitzer et al., 2006). This questionnaire is widely recognized for its effectiveness in identifying and measuring the severity of GAD symptoms. The Arabic version of this questionnaire has been endorsed by the Saudi Arabian Ministry of Health as a means of self-assessment for anxiety levels (Ministry of Health, 2024). Each item in the GAD-7 questionnaire evaluates different indicators of GAD, with respondents rating their frequency on a scale from 0 (not at all) to 3 (nearly every day). The total score ranges from 0 to 21, with higher scores indicating more severe anxiety. Anxiety severity is classified as follows: scores of 0–4 indicate minimal anxiety, 5–9 suggest mild anxiety, 10–14 represent moderate anxiety, and scores of 15–21 indicate severe anxiety. For analytical purposes, anxiety levels were grouped into two categories: minimal to mild anxiety or the presence of anxiety (moderate to severe), using a threshold score of ≥10 (Löwe et al., 2008).

### 2.7 Self-esteem

The evaluation of self-esteem utilized the validated Arabic version of a standardized single-item scale, where participants rated their agreement with the statement “I have high self-esteem.” This scale employed a 5-point Likert scale ranging from “not at all true of me” to “very true of me” (Fekih-Romdhane et al., 2023). The scoring system ranged from 1 to 5, where 1 indicated “not at all true of me,” 2 represented “rather not true of me,” 3 denoted “some part true of me,” 4 signified “true of me,” and 5 indicated “very true of me.” Participants selected the response option that best corresponded to their agreement or disagreement with the statement. A higher score indicated higher self-esteem, while a lower score indicated lower self-esteem. Responses were categorized into two groups: “Low self-esteem” (including responses 1 to 3) and “High self-esteem” (including responses 4 and 5) (Conner and Norman, 2017).

### 2.8 Perceived quality of life

Participants' perceived quality of life was assessed using a standardized single-item question derived from the Arabic version of the World Health Organization Quality of Life (WHOQOL) questionnaire. Respondents were asked, “How would you rate your quality of life?” and were provided a 5-point Likert scale to indicate their subjective evaluation, ranging from “very poor” to “very good (Ohaeri and Awadalla, 2009). This enabled the consistent quantification and analysis of the participants' self-reported quality of life as part of the broader survey data collection. Responses were dichotomized into two categories: “Low perception of quality of life” (including responses 1 to 3) and “High perception of quality of life” (including responses 4 and 5) (Conner and Norman, 2017).

### 2.9 Health satisfaction

Participants' level of health satisfaction was evaluated using a standardized single-item question derived from the validated Arabic version of the WHOQOL questionnaire: “How satisfied are you with your health?” Respondents were presented with a 5-point Likert scale to indicate their level of health satisfaction, with options ranging from “very dissatisfied” to “very satisfied” (Ohaeri and Awadalla, 2009). Responses were categorized into two groups: “Low health-satisfaction” (including responses 1 to 3) and “High health-satisfaction” (including responses 4 and 5) (Conner and Norman, 2017).

### 2.10 Dietary habits

Dietary habits were assessed using a short questionnaire aimed at assessing food intake in Qatar (Donnelly et al., 2018), a neighboring Arab country with similar characteristics to the KSA. From that questionnaire, 5 questions were adopted by the present study to evaluate the frequency of consuming specific dietary habits, involving the daily intake of vegetables, fruits, snacks, fast food, soft drinks and breakfast. Participants responded to questions about how often they consumed specific food items, with options ranging from “never” to “once or more daily”. For analytical purposes, the response options were consolidated into two categories: daily consumption vs. less than daily consumption.

### 2.11 Statistical analysis

The statistical analysis was conducted using IBM SPSS Statistics software, version 29.0 (Armonk, NY, USA: IBM Corp). Continuous variables were summarized as means and standard deviations, while categorical variables were reported as frequencies and percentages. Multivariate linear regression analysis was used to investigate the associations between the SREB score and other independent variables. Standardized Beta coefficients were calculated to estimate the magnitude of the effect of each independent variable on the SREB score, while accounting for the influence of the other variables included in the model. Multivariate binary logistic regression analysis was performed to examine the predictive factors associated with the high capacity for SREB. The odds ratio (OR) was used to estimate the likelihood of other variables predicting a high capacity for SREB. Both the linear regression and logistic regression analyses included the calculation of a 95% confidence interval (CI) for the estimated coefficients. The 95% CI represents the range of values within which the true population parameter is expected to lie with 95% confidence. Statistical significance was defined as a *p* < 0.05. To improve the clarity and coherence of the writing, the ChatGPT model (GPT-4, OpenAI, October 2023) was employed. This generative AI technology facilitated the enhancement of linguistic precision throughout the article.

## 3 Results

**Figure 1:**
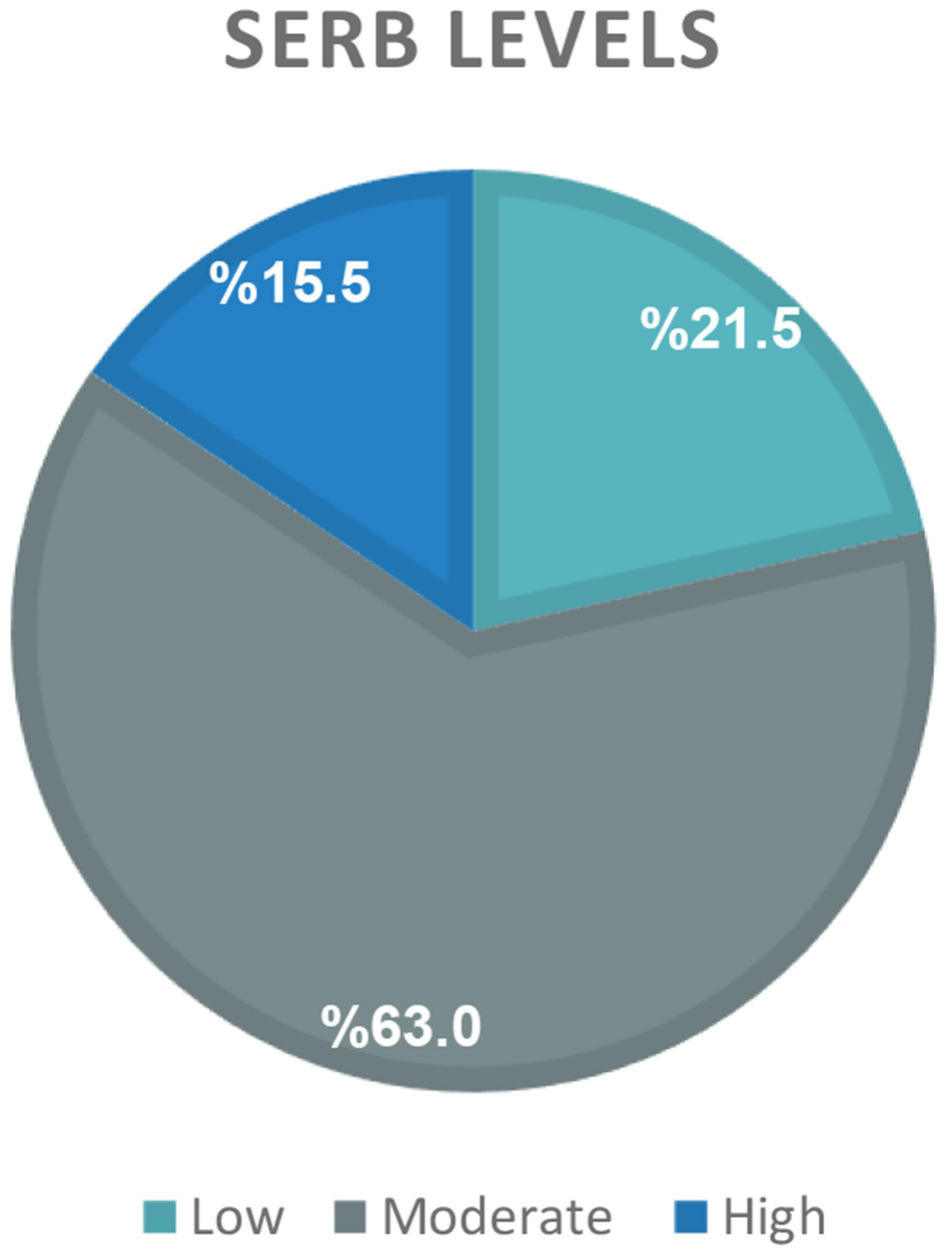
Distribution of self-regulation of eating behavior (SREB) levels.

**Figure 2:**
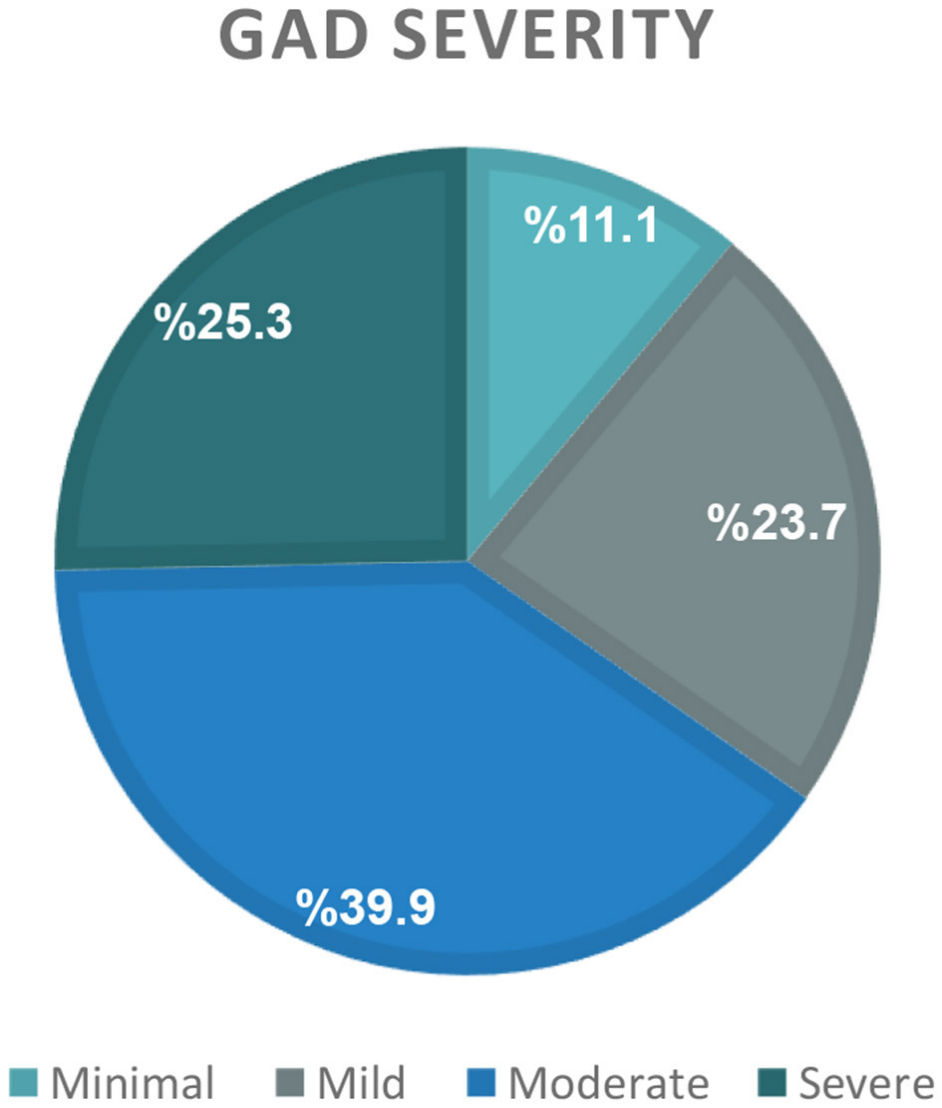
Generalized anxiety disorder (GAD) severity distribution.

The current study enrolled a total of 651 Saudi Arabian participants, with a mean age of 26.0 ± 6.0 years. Descriptive data are presented in Tables 1, 2 as well as Figures 1, 2. Females comprised the majority of the sample, accounting for 93.2% of the participants. Approximately one-third of the participants had a body weight that was higher than the healthy range. Specifically, 20.7% were with overweight, and 14.1% were with obesity (Table 1). High self-regulation of eating behavior (SREB) was observed only in 15.5 % of the participating sample (Figure 1). Nearly two-thirds of the participants were affected by generalized anxiety disorder (GAD), indicated by scores of 10 points or greater on the scale. Within this group, 39.9% exhibited moderate levels of anxiety, and 25.3% experienced severe levels of anxiety (Figure 2). With regards to dietary habits, a very low percentage, compromising only 8.8%, reported consuming fruits on a daily basis. Daily vegetable intake was also low, with just 19.7% of the participants reported eating vegetables daily. Conversely, a much higher proportion, close to one-third of the sample, indicated that they consumed snack foods such as chocolate and chips on a daily basis (Table 2).

**Table 1:** Sociodemographic characteristics of study participants.

**Variable**	***n* = 651**
**Age, years**	26.0 ± 6.0
**Sex**, ***n*** **(%)**	
Male	44 (6.8)
Female	607 (93.2)
**Education**, ***n*** **(%)**	
With university degree	285 (43.8)
Without university degree	366 (56.2)
**BMI, kg/m** ^**2**^	23.7 ± 5.3
**Weight status**, ***n*** **(%)**	
Underweight	106 (16.3)
Normal	318 (48.8)
Overweight	135 (20.7)
Obesity	92 (14.1)
**Self-esteem score**	3.9 ± 0.9
**Self-esteem classification**, ***n*** **(%)**	
Low	38 (5.8)
High	613 (94.2)
**Perceived quality of life score**	3.8 ± 0.8
**Perceived quality of Life classification**, ***n*** **(%)**	
Low	34 (5.2)
High	617 (94.8)
**Health Satisfaction score**	3.4 ± 1.0
**Health Satisfaction classification**, ***n*** **(%)**	
Low	121 (18.6)
High	530 (81.4)

All data are mean and standard deviation unless indicated.

**Table 2:** Dietary habits of study participants.

**Variable**	***n* = 651; *n* (%)**
**Daily intake of fruits**	
No	594 (91.2)
Yes	57 (8.8)
**Daily intake of vegetables**	
No	523 (80.3)
Yes	128 (19.7)
**Daily intake of breakfast**	
No	404 (62.1)
Yes	247 (37.9)
**Daily intake of snacks**	
No	449 (69.0)
Yes	202 (31.0)
**Daily intake of soft drinks**	
No	590 (90.6)
Yes	61 (9.4)

The results of multivariate linear and logistic regression analyses are presented in Tables 3, 4, respectively. The linear regression analysis revealed that higher SREB scores were significantly associated with lower scores of GAD (β = −0.13, 95% CI; −0.12 to −0.03; *p* < 0.001). This negative association suggests that greater symptomatology of generalized anxiety disorder is related to lower levels of self-regulation of eating behavior among the study participants. In other words, individuals exhibiting more severe symptoms of generalized anxiety disorder tend to display poorer self-regulation of their eating behavior. Conversely, self-esteem was found to be positively associated with SREB scores (β = 0.11, 95% CI; 0.11 to 0.56; *p* = 0.003) (Table 3). Furthermore, health satisfaction was positively associated with a nearly 5-fold increase in the likelihood of exhibiting elevated SREB (OR = 4.88, 95% CI; 1.67 to 14.19; *p* = 0.004) (Table 4).

**Table 3:** Factors associated with self-regulation of eating behavior score.

**Variable**	**β**	**95% CI**	***P***
Age, years	0.10	(−0.00,0.04)	0.09
Sex, female	−0.00	(−0.81,0.82)	0.9
Education, with a university degree	−0.11	(−1.07,−0.20)	**0.004**
BMI, kg/m	−0.10	(−0.08,−0.00)	**0.01**
Self-esteem score	0.11	(0.11,0.56)	**0.003**
Perceived quality of life score	0.03	(−0.18,0.37)	0.5
Health satisfaction score	0.17	(0.23,0.68)	**<0.001**
GAD score	−0.13	(−0.12,−0.03)	**<0.001**
Vegetables daily intake	0.10	(−0.00,1.11)	0.05
Fruits daily intake	0.10	(−0.11,1.45)	0.09
Breakfast daily intake	0.14	(0.41,1.27)	**<0.001**
Snacks daily intake	−0.13	(−1.24,−0.36)	**<0.001**
Soft drinks daily intake	0.02	(−0.53,0.87)	0.6

Data are presented as standardized beta coefficient (β) with 95% confidence intervals (CIs) using a multivariate linear regression analysis. Bold values indicates the p-value at the level of significance.

**Table 4:** Factors associated with high self-regulation of eating behavior.

**Variable**	**OR**	**95% CI**	***P***
Age, years	1.03	(0.99,1.05)	0.08
Sex, female	0.46	(0.20,1.02)	0.05
Education, with a university degree	0.57	(0.33,0.95)	**0.03**
Overweight/obesity	0.55	(0.31,0.96)	**0.03**
High self-esteem	2.85	(0.61,13.11)	0.1
High perceived quality of life	0.97	(0.18,4.99)	0.9
High health satisfaction	4.88	(1.67,14.19)	**0.004**
Moderate/severe GAD	1.19	(0.71,1.98)	0.5
Daily intake of vegetables	1.68	(0.94,2.97)	0.07
Daily intake of fruits	2.90	(1.44,5.84)	**0.003**
Daily intake of breakfast	1.64	(1.01,2.63)	**0.04**
Daily intake of snacks	0.53	(0.30,0.92)	**0.02**
Daily intake of soft drinks	1.66	(0.75,3.68)	0.2

Data are presented as Odds Ratios (ORs) with 95% confidence intervals (CIs) using a multivariate logistic regression analysis. Bold values indicates the p-value at the level of significance.

Regarding anthropometric and dietary factors, an inverse association was observed between SREB scores and BMI (β = −0.10, 95% CI; −0.08 to −0.00; *p* = 0.01), such that higher SREB was linked to lower BMI. Specifically, this finding suggests that for every one-unit increase in BMI, SREB scores decrease by 0.10 units (Table 3). According to the logistic regression analysis, the likelihood of achieving a high level of SREB is reduced by 45% among participants with overweight or obesity compared to those with normal or underweight status (OR = 0.55, 95% CI; 0.31 to 0.96; *p* = 0.03), (Table 4). Conversely, the reciprocal of this odds ratio (1/0.55 ≈ 1.82) implies that participants classified as normal weight or underweight are ~1.82 times more likely to have high SREB than their counterparts with overweight or obesity.

Interestingly, the daily intake of fruits was positively associated with a higher likelihood of having a high SREB. Specifically, the probability of having a high level of SREB was nearly three times higher for participants who consumed fruits every day compared to those who did not (OR= 2.90, 95% CI; 1.44 to 5.84; *p* = 0.003). Additionally, daily breakfast intake was associated with a 64% increase in the likelihood of having a high SREB (OR= 1.64, 95% CI; 1.01 to 2.63; *p* = 0.04). Conversely, the daily snack consumption was associated with reduced capacity for SREB. Specifically, participants who reported eating snacks every day were 47% less likely to exhibit high SREB levels compared to those who did not (OR = 0.53, 95% CI; 0.30 to 0.92; *p* = 0.02) (Table 4).

## 4 Discussion

The relationship between self-regulation of eating behavior (SREB), dietary habits, and mental health is a growing area of scientific interest (Schmalbach et al., 2021; Barak et al., 2021; Annesi, 2021; Dakanalis et al., 2023). Within the context of Saudi Arabia, this research represents the initial exploration of SREB, with anxiety levels and dietary behaviors examined as predictive factors. According to the findings of the present study, the prevalence of high SREB was found to be relatively low, accounting for only 15.5% of the studied population. This finding is consistent with the only other published study on SREB in the KSA, which reported a prevalence rate of 17.7% (Al-Hazmi and Noorwali, 2023). Notably, the investigation indicated that higher levels of SREB were linked to lower BMI values among the Saudi population. This inverse relationship between SREB and BMI aligns with the observations reported in studies from the United Kingdom (Kliemann et al., 2016), United States of America (Graff et al., 2021), and Germany (Schmalbach et al., 2021). Such association may be attributed to the increased likelihood of individuals with stronger self-regulatory eating habits to embrace mindful and healthier eating behaviors, thereby resulting in better weight management and lower BM (Román and Urbán, 2019). Accordingly, it can be concluded that high capacity for SREB can be considered a protective factor against the development of overweight and obesity. Hence, it is important to consider strategies aimed at enhancing SREB as they could prove beneficial in addressing the escalating rates of obesity within the KSA.

Interestingly, the current study detected an inverse association between higher scores of SREB and lower scores of GAD. It is suggested that individuals with a greater capacity for self-regulating their eating behaviors tend to also experience fewer symptoms of generalized anxiety. This is in line with the findings of a study conducted in the USA which found that higher capacity for SREB is associated with a reduction in anxiety levels among university students (Graff et al., 2021). Another study from Netherlands found that higher capacity for SREB was linked to improved overall wellbeing in young adults (Telman, 2023). These findings hold significance in the KSA context, particularly considering the widespread prevalence of anxiety reported among the population (Altwaijri et al., 2020). Although the mechanism underlying the negative association between SREB and anxiety is not yet clear, it is hypothesized that SREB could be associated with healthy dietary choices that may improve mental health and overall wellbeing. Previous reviews have indicated that maintaining healthy dietary patterns is linked to favorable mental health outcomes, whereas adopting less healthy dietary patterns may have detrimental effects on an individual's psychological wellbeing (Głabska et al., 2020; Ventriglio et al., 2020; Teasdale et al., 2019; Grajek et al., 2022; Collins et al., 2022).

The exact processes through which healthy dietary choices may impact mental health are also not yet fully understood. However, it is proposed that healthy food choices rich in substances like antioxidants and vitamins may influence pathways related to inflammation regulation, and oxidative stress, both of which are factors linked to psychological wellbeing (Suárez-López et al., 2023; Godos and Grosso, 2021). The present study's findings offer support for such proposed hypotheses. For instance, there was a notable positive association between a higher likelihood of having high SREB and maintaining healthy dietary habits. Specifically, participants who consume fruits on daily basis have a nearly threefold higher probability of having a high level of SREB compared to those who do not. This is consistent with previous studies that found a positive association linking the high intake of fruits and vegetables to high SREB (Kliemann et al., 2016, 2018). Additionally, the present study found that individuals who consume breakfast daily show a nearly 64% higher probability of having a high level of SREB compared to those who skip breakfast. It is expected that individuals who regularly consume breakfast may be more likely to make healthier food choices throughout the day, contributing to higher capacity for SREB.

Finally, it was found that higher SREB is associated with higher self-esteem and higher health satisfaction. Mechanisms through which self-esteem and health satisfaction may lead to higher levels of SREB likely involve multiple pathways. For instance, individuals with high self-esteem and health satisfaction tend to have greater confidence in their abilities to maintain their wellbeing. This confidence may translate into healthier dietary choices and more disciplined eating habits. Additionally, positive self-esteem and satisfaction with one's health promote feelings of empowerment and emotional resilience, which could reduce the likelihood of impulsive or emotional eating (Verstuyf et al., 2012; Chammas et al., 2024; Guertin and Pelletier, 2023). Overall, fostering positive self-esteem and promoting health satisfaction may facilitate improved self-regulation of eating behavior and contribute to overall wellbeing.

However, it is important to note that the relationship between SREB and self-esteem, dietary habits and anxiety might be bidirectional (Dohle et al., 2018; Polivy and Herman, 2005), and further investigations are warranted to better understand these associations. Moreover, it is worthy to acknowledge the potential role of mediating and moderating factors that may influence the relationships between SREB and other variables, which were not investigated in the present study. For instance, it may be recommended to consider the influence of socioeconomic status and physical activity level, on such associations in future research. Identifying the potential mechanisms and boundary conditions that shape the links between self-regulation of eating and related psychosocial factors is crucial for developing a more comprehensive understanding of this complex phenomenon.

Given the important role of SREB in addressing obesity and its potential association with anxiety, it is crucial to consider the key factors that influence SREB levels. The findings of this study highlight the importance of healthy dietary habits, particularly the daily consumption of fruits and breakfast, as well as the role of high self-esteem, in shaping SREB. These insights can inform the development of targeted interventions and public health strategies to enhance SREB and address the related health challenges in the KSA.

### 4.1 Strength and limitations

The key strength of this study is that it is the first to explore the associations between SREB, mental health, and dietary habits among the Saudi population. However, it has several limitations that should be considered when interpreting the findings. The cross-sectional design of the study restricts the ability to infer causal relationships between the variables. Future longitudinal investigations are needed to establish the directionality of the associations observed in this research. Additionally, the reliance on self-reported data for dietary habits, anthropometric measurements and SREB may be subject to recall bias and social desirability bias, potentially affecting the accuracy of the information collected. This limitation should be addressed in subsequent studies by incorporating more objective measures of these variables. Finally, the present investigation did not explore the potential mediating or moderating factors that could influence the relationships between SREB, mental health, and dietary habits. Incorporating an examination of intermediary variables could provide additional insights into the complex interplay between these constructs and inform the development of more targeted interventions.

## 5 Conclusions

The present study suggests that a high capacity for self-regulation in eating may be beneficial in reducing obesity rates in the KSA and improving mental health among the Saudi population. The promotion of healthy dietary habits emerges as a promising strategy to enhance self-regulatory eating behaviors. However, it is crucial to consider potential bidirectional associations and the influence of mediators and moderators, such as socioeconomic status and physical activity. Therefore, further research is needed to gain a better understanding of these associations and to inform appropriate interventions aimed at increasing SREB among the Saudi population, thereby improving both physical and mental wellbeing among the population living in the KSA.

## Data Availability

The raw data supporting the conclusions of this article will be made available by the authors, without undue reservation.
